# Spatial Heterogeneity of Soil Bacterial Community Structure and Enzyme Activity along an Altitude Gradient in the Fanjingshan Area, Northeastern Guizhou Province, China

**DOI:** 10.3390/life12111862

**Published:** 2022-11-12

**Authors:** Yuangui Xie, Lanyue Zhang, Juncai Wang, Meng Chen, Jiming Liu, Shengyang Xiao, Xiu Tian, Tingting Wu

**Affiliations:** 1 College of Forestry, Guizhou University, Guiyang 550000, China; 2 Institute of Mountain Resources, Guizhou Academy of Sciences, Guiyang 550000, China; 3 Guizhou Academy of Forestry, Guiyang 550000, China

**Keywords:** Fanjingshan area, bacterial community, soil enzymes, altitude, spatial differences

## Abstract

Changes in altitude can cause regional microclimate changes, leading to the spatial heterogeneity of environmental factors and soil bacteria. However, the internal driving process and mechanism remain unclear. Here, we selected Fanjingshan, a typical nature reserve in the subtropical region of south China with a clear altitudinal belt, to reveal the response mechanisms of microbial populations with altitude changes. We examined the physiochemical and biological properties (pH and soil enzyme activities) of 0~10 cm soil layers, soil bacterial diversity, and community structure across the 2.1 km belt (consisting of six altitude ranges). Our results showed that soil pH was highest at the altitude range below 900 m and decreased with altitude thereafter. Soil enzyme activities showed an overall decreasing trend with altitude rising. The soil sucrase and catalase activity was highest (48.35 mg.g^−1^.d^−1^ and 23.75 µmol.g^−1^, respectively) at altitudes below 900 m; the soil urease activity was highest (704.24 µg.g^−1^.d^−1^) at 900~1200 m; and the soil acid phosphatase activity was highest (57.18 µmol.g^−1^) at 1200~1500 m. In addition, the soil bacterial community diversity showed a linear increasing trend, with the maximum abundance at 1500~1800 m. Soil pH was correlated with enzyme activity and bacterial community composition and structure, and the correlation was the strongest between pH and the distribution of bacterial diversity at altitudes below 900 m. Overall, soil enzyme activities and soil bacterial diversity showed spatial heterogeneity along the altitude gradient, and their community structure and composition were affected by altitude as a result of changes in soil physicochemical factors. This study provides a better and deeper understanding of the spatial succession of soil in the Fanjingshan area and the distribution pattern of soil microorganisms in central subtropical mountain ecosystems.

## 1. Introduction

Soil microbes and enzymes are important components in soils, which play a crucial role in ecosystems [1,2]. They act not only as regulators of ecosystem functions but also as major drivers of nutrient transformation and material cycling in ecosystems [3,4]. Soil microbial diversity, community structure stability, and enzyme activity reflect soil nutrient status [5], and thus play an important role for a plant’s growth and its adaptive capacity to the environment. The study of soil microorganisms and enzyme activities is important for understanding soil fertility, soil environment, soil nutrient effectiveness for plants, and soil nutrient transformation and cycling [6]. Soil bacteria are the main group of soil microorganisms. Their diversity and community turnover are susceptible to environmental factors, and can be used as effective indicators of the status of environmental variables [7]. Investigating the variation of soil bacteria and enzyme activities along an altitude gradient could reveal the vertical variation pattern of soil.

Spatial heterogeneity is found among ecological factors in the vertical direction in mountain ecosystems [8]. For example, the changes in altitude gradients lead to changes in various environmental factors such as temperature, light, and moisture. Conversely, the microclimate and soil physicochemical and biological characteristics of mountainous regions have gradient changes [9]. It is important to investigate the relationship between them in order to study the regional spatial succession pattern. Hence, the study of the association mechanism between soil microbial diversity and soil physicochemical factors at different altitude gradients has become a research topic. Many studies have shown that soil microbial structure and enzymatic activity varied with environmental factors along an altitude gradient. For example, Ma et al. [10] found that soil bacterial populations in the Guanshan Nature Reserve showed a monotonic decrease with increasing altitude, and the abundance-dependent phylogenetic index did not vary along the altitude gradient. Bryant et al. [11] also showed a monotonous decrease in bacterial community diversity from low to high altitude. Wu et al. [12] investigated the changes in soil microbial functional community diversity along an altitude gradient and their influencing factors on Mount Wuyi, and found significant differences in the functional diversity of soil microbial communities with altitude. This variability was affected by a combination of factors such as biomass, forest litter, soil nutrients, micro fauna, and plant roots [12]. Tang et al. [13] applied structural equation modeling (SEM) to study the regulation of soil microorganisms by biotic and abiotic factors, and found that soil pH had the greatest direct effect on soil microbial diversity and community structure, while altitude was the main controlling factor. Although some studies have also shown that soil microorganisms were not significantly influenced by progression along an altitude gradient [8], soil microbial biomass was highly significantly and positively correlated with soil enzyme activity [14]. In addition, Siles et al. [15] showed a significant increase in soil microbial activity and abundance with elevation in the Italian Alps. It is clear that soil microbial and enzymatic activities vary with altitude in mountain ecosystems of different regions as a result of differences in zonal or non-zonal hydrothermal conditions and the ecosystem types in the basal zone.

Mount Fanjingshan, the main peak of the Wuling Mountains, is the earliest area south of the Yellow River in China to be raised from the ocean to land [16], with an altitude range of 450~2572 m [17]. The forest cover ratio is 95%, and it is a forest ecosystem with a large and continuous distribution area, high levels of ecological preservation, clear altitudinal belt, and very rich biological resources among the mountain forests of the same latitude in the central subtropical region [18]. It has been shown that the distribution of AM fungi, orchids, leafhoppers, and other plant and animal species in Fanjingshan is influenced by vegetation type, soil, altitude, and other factors [19,20]. However, to date, there has been no research on soil enzyme activity and on the relationship between soil microbial diversity and enzyme activity along an altitude gradient in the Fanjingshan area. Therefore, we hypothesized that soil physicochemical and biological properties and microbial diversity changed significantly with altitude in this region.

This study was conducted in the Fanjingshan area to collect soil samples at six different altitudes, and to determine the diversity and community structure of microbial bacteria and enzyme activities in soils using high-throughput sequencing and enzyme-labeled microplate methods. The aims of this study were to (1) clarify the characteristics of soil physicochemical and biological properties and enzyme activities in response to different altitude gradients in Fanjingshan; (2) reveal the changes of soil microbial diversity and community structure in response to different altitude gradients; and (3) analyze the correlation between soil physicochemical and biological properties and the distribution of microbial bacteria. In short, the results of this study will provide a better understanding of the spatial succession pattern of soil in Fanjingshan and the distribution pattern of soil microorganisms in central subtropical mountain ecosystems.

## 2. Research Area and Methods

### 2.1. Research Area

**Figure 1:**
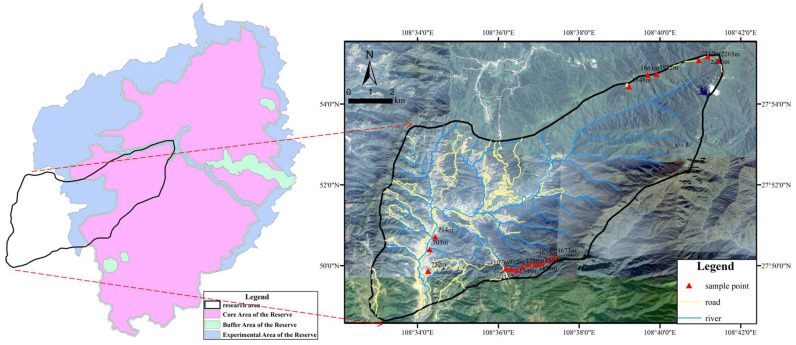
Geographical location map of research area and sample points.

Fanjingshan Mountain, situated in the northeast part of Guizhou Province, is one of China’s national nature reserves established in 1978. There are rare species such as the Guizhou golden monkey, the Fanjingshan fir, and the dove tree. It has a total area of 567 km^2^, a heritage site area of 402.75 km^2^, and a buffer zone of 372.39 km^2^. Against a background of the commonplace destruction of the global natural environment, Fanjingshan Mountain is a natural complex subtropical forest ecosystem that is well protected. Fanjingshan is scientifically important and regarded as an ideal base for scientific research and education (The group of the Scientific survey of the Fanjingshan Mountain 1982 [21]). Our research area has an area of 87.13 km^2^ and is located at 108°33′–108°41′ E and 27°48′–27°55′ N (Figure 1). It has the characteristics of a humid climate in the central subtropical monsoon zone, with an average annual temperature of 5~17 °C, an annual precipitation of 1100~2600 mm, and a relative humidity of more than 80% [22]. The most widely distributed soil types are yellow loam and dark yellow-brown loam. The vegetation along the altitude gradient shows obvious spatial distribution characteristics because of the vertical variation of environmental factors such as altitude and climate from the base to the top of the mountain. The forest types of the mountain from the bottom to the top are as follows: coniferous forest, conifer-broadleaf mixed forest, evergreen broadleaf forest, deciduous broadleaved mixed forest, and evergreen and deciduous broadleaved mixed forest, with mossy dwarf forest and alpine scrub at the top of the mountain (The group of the scientific survey of the Fanjingshan Mountain 1982 [21]).

### 2.2. Soil Sample Collection

Following the Niuwei River in the research area, research sample plots were set up along an altitude gradient. The altitude gradient was set at intervals of 300 m and split into six altitude ranges from bottom to top (below 900 m, 900~1200 m, 1200~1500 m, 1500~1800 m, 1800~2100 m, and 2100~2400 m) (Figure 1). A total of 10 m × 20 m quadrat was selected by the minimum area method within each altitude range. In each quadrat, three sampling points with similar slope-direction, slope, and surface vegetation were selected (Table 1).

The sampling was carried out by removing the litterfall from the surface layer of the forest and using a sterile shovel and hoe disinfected with alcohol to dig a soil profile vertically at the center of each sample site. The depth of the profile was determined according to the depth of the soil so that the excavation reached the bedrock. The same layer of soil was mixed as one sample. Approximately 2 kg of soil was removed at each sampling point. The collected soil sample was divided into two parts. The first part was placed into sterilized plastic bags, then quickly stored in ice boxes and brought back to the laboratory. In the laboratory, it was placed in a −80 °C refrigerator for use in the high-throughput sequencing of soil microorganisms. The second part of the soil sample (~1 kg) was placed in a cloth bag numbered without contaminants and brought back to the laboratory under warm ambient conditions (25 °C). After it had dried naturally, one half of it was taken by the diagonal quartering method. This part was ground and sieved to remove non-soil materials such as plant residues and large gravels, while contamination by dust and acids or bases was avoided. After this treatment, it was used for pH and enzyme activity determination. Three replicate samples were collected from each sample plot.

### 2.3. Determination Methods

#### 2.3.1. PH and Soil Enzyme Activity Determination

This study tested factors related to microbial distribution and metabolism, including pH and four enzyme activities (soil urease, soil catalase, soil sucrase, and soil acid phosphatase abbreviated as S-UE, S-CAT, S-SC, and S-ACP, respectively). Soil pH was determined by CO_2_-removed distilled water extraction. Ten grams of air-dried soil sample were extracted with 25 mL of distilled water for 30 min, and then a precision pH meter (phs-3c) was used for direct determination. Soil enzymes were determined by the microplate method with an enzyme marker, referring to the kit instructions (Beijing Solarbio Science & Technology Co., Beijing, China).

S-UE activity was determined by the spectrophotometric method at 578 nm based on the released ammonia after incubation of the toluene treated soil samples with a urea solution at 37 °C for 24 h [23]; S-CAT activity in the soil was determined by the KMnO_4_ method whereby the residual H_2_O_2_ added in soil was back-titrated by 0.1 M KMnO_4_ [24]; S-SC activity was determined by incubating soil in 8% sucrose solution and phosphate buffer (pH 5.5) at 37 °C for 24 h and then measuring the released reducing sugars, such as glucose, at 508 nm with ammonium molybdate colorimetry [25]; and S-ACP activity was measured by the disodium phenyl phosphate colorimetric method and the amount of phenol released after 24 h culture at 37 °C was assayed colorimetrically at 660 nm [26], respectively.

#### 2.3.2. DNA Extraction, NovaSeq Sequencing, and Bioinformatics Analysis

Total bacterial DNA was isolated from 0.50-g soil per samples area and total DNA was extracted by the CTAB method. Each sample area had three biological replicates, for a total of 18 soil samples. The quality of extracted DNA was detected by 1.2% agarose gel electrophoresis, and the V3 + V4 variable region of the bacterial 16 S rRNA gene was amplified by PCR with the common primer pair 341F (5′-CCTAYGGGRBGCASCAG-3′) and 806R (5′-GGACTACNNGGGTATCTAAT-3′) primers [27]. All PCR reactions were conducted with 15 μL of Phusion^®^ High-Fidelity PCR Master Mix (New England Biolabs, Ipswich, MA, USA), 2 μM of forward and reverse primers, and 10 ng template DNA. Standard curves were generated using a series of 10-fold dilutions to generate a concentration range of 10^2^ to 10^8^ copies per assay. The PCR amplification procedure was as follows: 98 °C pre-denaturation for 1 min, 30 cycles (with these conditions: 98 °C at 10 s; 50 °C for annealing at 30 s; 72 °C for elongation at 30 s; and 72 °C continue to extension at 5 min and end). PCR products mixed with the same volume of 1 loading buffer (contained SYB green) were detected and purified by electrophoresis using a 2% agarose gel. Product purification was conducted using Qiagen’s Gel Recovery Kit. The library was constructed using an Illumina TruSeq DNA PCR-Free Sample Preparation Kit (Illumina, San Diego, CA, USA), and the constructed library was quantified and tested by Qubit. After quantification, NovaSeq 6000 PE250 was used for on-machine sequencing. Quality filtered data were analyzed using the “Atacama soil microbiome tutorial” “https://docs.qiime2.org (accessed on 1 January 2019)” in Qiime2, with reference to the method described by Bokulich [28].

### 2.4. Data Analysis

One-way analysis of variance (ANOVA) and a multiple comparison Tukey’s test (*p* < 0.05) for significant differences between experimental data were performed using IBM SPSS version 22.0. Graphs of pH and soil enzyme data were constructed and plotted using Origin 2018. The sequences with 99% or more similarity were selected to construct the species classification information table. Alpha diversity indices (including observed operational taxonomic units (OTUs), Chao1, Shannon indices, and Faith’s phylogenetic diversity (PD)) were selected to evaluate the degree of soil microbial diversity. Beta diversity indices (including Bray–Curtis, unweighted UniFrac, and weighted UniFrac) were used to assess the taxonomic and phylogenetic variability of soil microbial communities at different elevations. Differences in community abundance were tested using linear discriminant analysis (LDA), effect size (LEfSe) cladogram, and LDA was used to find the characteristic microorganisms for each group. Principal co-ordinate analysis (PCoA) and non-metric multidimensional scaling (NMDS) plots were presented using the R package “vegan”. A redundancy analysis (RDA) was used to characterize the potential associations of microbial communities with soil pH and soil enzymes. In addition, a Spearman correlation analysis was used to reveal the relationship between soil enzymes and soil microorganisms. The analysis of microbial community structure and composition was conducted using the Wekemo Bioincloud (https://www.bioincloud.tech, accessed on 19 October 2022).

## 3. Results

### 3.1. Changes in PH and Soil Enzymes along the Altitude Gradient

**Figure 2:**
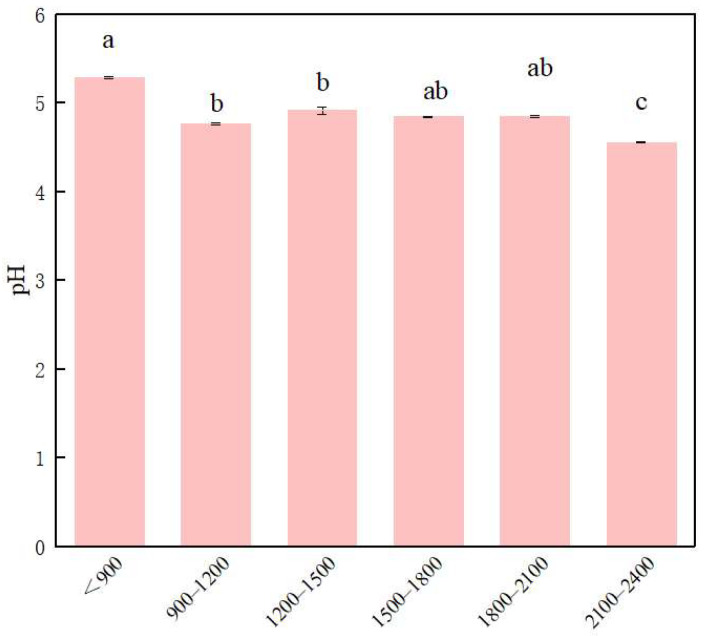
Changes in pH along the altitude gradient (mean ± SD). Different lowercase letters indicate significant differences (*p* < 0.05) among different altitude gradient.

The soil pH showed an overall decreasing trend with increasing altitude (Figure 2). However, the variation was small, especially at 900~1200, 1200~1500, 1500~1800, and 1800~2100 m, in which the pH value remained almost unchanged, and there was no significant difference between these altitude gradients. The pH value at 900~1200 m was 0.52 lower than that at below 900 m. Moreover, the pH value at altitudes below 900 m was significantly different from that at altitudes above 2100 m. This indicates that soil pH is influenced by altitude gradient change in vertical space.

**Figure 3:**
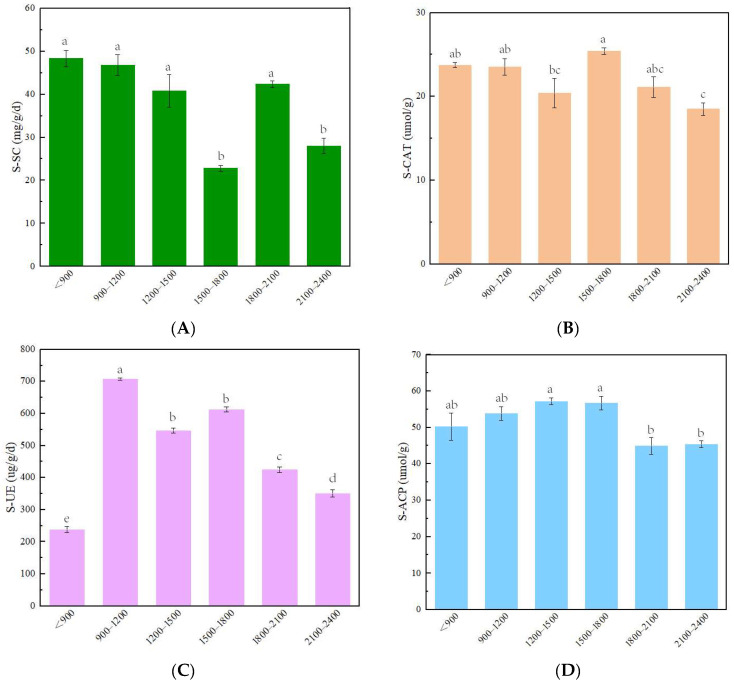
Changes in soil enzymes along the altitude gradient ((**A**) changes in S-SC, (**B**) changes in S-CAT, (**C**) changes in S-UE, (**D**) changes in S-ACP). Different lowercase letters indicate significant differences (*p* < 0.05) among different altitude gradient.

The four soil enzyme activities varied with the altitude gradient in different patterns (Figure 3). The S-SC activity ranged from 22.84 to 48.35 mg.g^−1^.d^−1^ and was lower for 1500~1800 m and 2100~2400 m, which was significantly different from all other altitude ranges. The S-CAT activity ranged from 18.52 to 25.40 µmol.g^−1^, with the highest activity observed at 1500~1800 m, which showed significant differences from the lowest activity, which was observed at 2100~2400 m. The S-UE activity ranged from 237.78 to 707.24 µg.g^−1^.d^−1^, with the lowest activity observed at the altitudes below 900 m. The S-UE activity in each altitude range was significantly different from the others, except for those at 1200~1500 and 1500~1800 m. The S-ACP activity ranged from 44.85 to 57.18 µmol.g^−1^ and showed an overall trend of increasing and then decreasing with increasing altitude; the activities at 1200~1500 and 1500~1800 m were both significantly different from those at 1800~2100 and 2100~2400 m. Overall, soil enzyme activity was spatially heterogeneous across the altitude gradient.

### 3.2. Changes in Soil Bacterial Community Composition and Species Differences among OTU Groups along the Altitude Gradient

**Figure 4:**
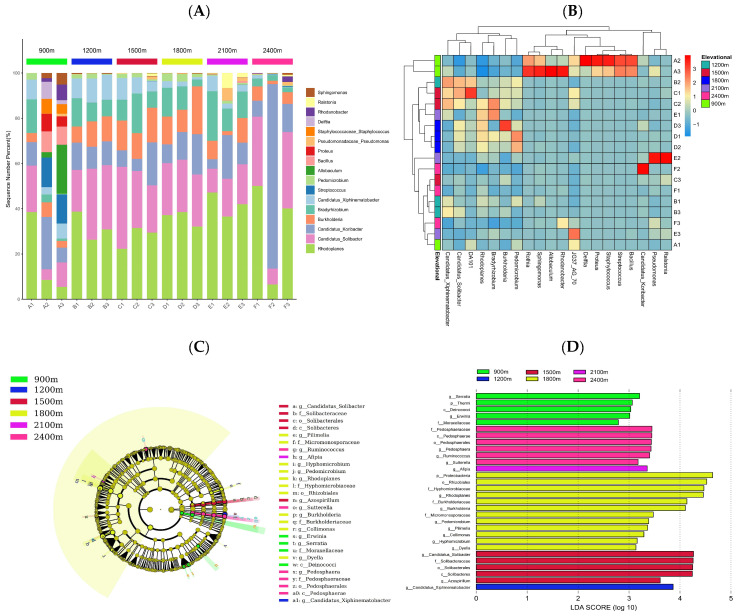
Changes in soil bacterial community composition and species differences among OTU groups along the altitude gradient ((**A**) taxonomic composition of soil bacteria along the altitude gradient, (**B**) a heat map reflecting the similarity of genera between different altitude gradients, (**C**) the evolutionary branch diagram of differential taxa, (**D**) main indicator microbial groups).

The top 20 most dominant genera were selected to determine the taxonomic composition of soil bacteria along the altitude gradient (Figure 4A). *Rhodoplanes*, *Candidatus solibacter*, *Candidatus koribacter*, and *Bukholderia* were the dominant genera, accounting for more than 80% of the sequence distribution bars. The abundances of *Bradyrhizobium* and *Candidatus xiphinematobacter* were at a relatively high level, while the abundances of *Sphingomonas* and *Ralstonia* were at a lower level. We observed that species richness was highest for altitudes below 900 and 2100~2400 m. Based on the Bray–Curtis similarity index, the abundances of the top 30 classified microbial genera were selected to build a heat map (Figure 4B). The clustering tree indicated that the altitude range of 1200~1500 m had high similarity with 900~1200, 1800~2100, and 2100~2400 m. The heat map showed that the abundances of the top 20 dominant genera decreased as the altitude increased. The LEfSe (Figure 4C,D) showed that a total of 30 differential taxa were identified (*p* < 0.05). At altitudes below 900 m, *Serratia*, *Thermi*, and *Deinococci* were the three most significant biomarkers; at 900~1200 m, *Candidatus xiphinematobacter* was the only significant biomarker; at 1 200~1500 m, *Candidatus solibacter* and *Solibacteraceae* were the two most significant biomarkers; at 1500~1800 m, *Proteobacteria*, *Rhizobiales*, and *Hyphomicrobiaceae* were the three most significant biomarkers; at 1800~2100 m, *Afipia* was the only significant biomarker; and at 2100~2400 m, *Pedosphaerae*, *Ruminococcus*, and *Sutterella* were the three most significant biomarkers.

### 3.3. Differences in Soil Microbial Diversity and Community Structure along the Altitude Gradient

**Figure 5:**
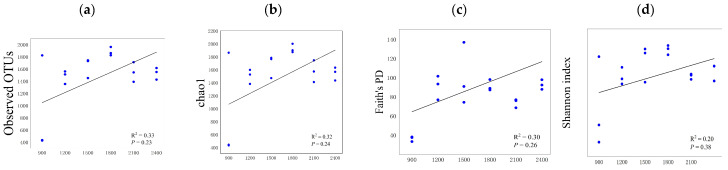
Changes in *Alpha* diversity of soil bacteria along the altitude gradient ((**a**) OTU abundance, (**b**) chao1 index, (**c**) Faith’s PD, (**d**) Shannon index).

The change in soil bacterial diversity in Fanjingshan was not significant (*p* < 0.01) along the altitude gradient (Figure 5). OTU abundance (ranging from 427 to 1961) and Chao1 index (ranging from 433 to 2000) both showed a linear increasing trend with increasing altitude, and they were both highest at 1500~1800 m. Similarly, Faith’s PD and Shannon index also showed a linear increasing trend, although the trend was relatively flat. For Faith’s PD, the highest difference was observed between the altitude range of below 900 m (SD = ±1.51) and that of 1200~1500 m (SD = ±9.41). For the Shannon index, there was no significant difference at any altitude range, and the highest value was observed at 1500~1800 m.

**Figure 6:**
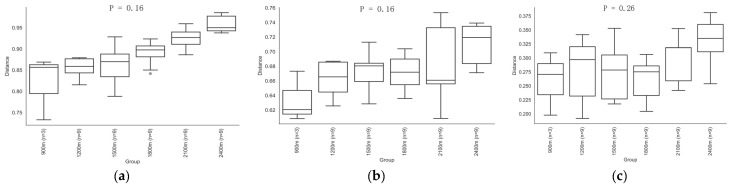
Box plot of Beta diversity of bacteria along the altitude gradient ((**a**) Bray–Curtis distances, (**b**) unweighted UniFrac distances, (**c**) weighted UniFrac distances).

The ANOSIM method was used to compare the similarity of soil bacterial composition and structure among different altitude gradients through a distance measure. As shown in Figure 6, there was no significant difference in altitude. The Bray–Curtis distance showed that the taxonomy and phylogeny of soil bacteria at 1500~1800 m was different from that at other altitude ranges. The unweighted and weighted UniFrac distances revealed no significant differences in taxonomic and phylogenetic structures among the six altitude gradients.

### 3.4. Effect of PH and Soil Enzyme on Microbial Community Structure

**Figure 7:**
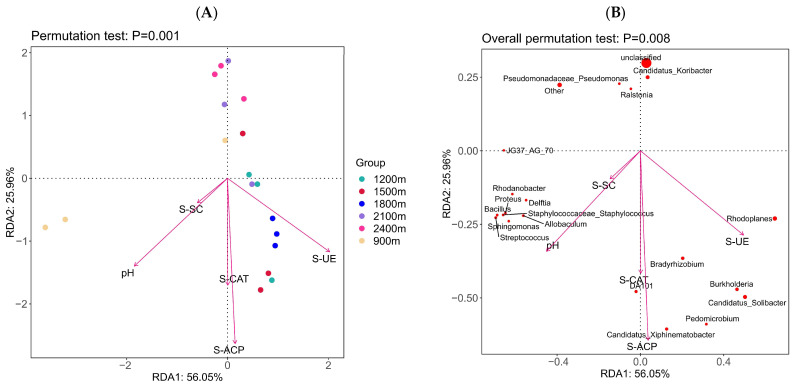
Results of RDA analysis based on linear model at genus level ((**A**) RDA ordination of soil factors and altitude, (**B**) RDA ordination of soil factors and microbial community structure).

The RDA based on linear modeling showed that the differences in soil bacterial community structure at different altitude gradients were significantly correlated with soil pH and soil enzymes (*p* < 0.01). As shown in Figure 7A, pH and S-SC were significantly positively correlated with the distribution of bacterial diversity at altitudes below 900 m, with pH showing the strongest correlation. S-CAT, S-ACP, and S-UE were significantly positively correlated with the distribution of bacterial diversity at altitude ranges of 900~1200, 1200~1500, and 1500~1800 m, and S-ACP was the most strongly correlated. At 1800~2100 and 2100~2400 m, the distribution of bacterial diversity showed no correlation with soil pH and soil enzymes. The RDA analysis of the top 20 classified microbial genera abundance with soil pH and soil enzymes (Figure 7B) showed that *Rhodoplanes*, *Bradyrhizobium*, and *Burkholderia* were positively correlated with S-CAT, S-ACP, and S-UE to different degrees, and were negatively correlated with pH and S-SC. Taxa such as *Delftia*, *Proteus*, and *Xiphinematobacter* showed positive correlations with pH and S-SC, and negative correlations with S-CAT, S-ACP, and S-UE. *Pseudomonadaceae* and *Ralstonia* showed no significant correlations with pH and soil enzymes. S-SC, S-CAT, S-ACP, and S-UE activities showed positive correlations with pH, with S-ACP showing the highest correlation.

## 4. Discussion

### 4.1. Variation of Soil Enzyme Activity with Altitude in Fanjingshan

Soil enzymes, as bioactive indicators for evaluating soil quality, are often affected by soil biochemical properties, soil hydrothermal conditions, soil biology, and vegetation types and diversity. These environmental factors change with altitude; thus, soil enzyme activity shows a distribution pattern along the altitude gradient [29]. In general, soil enzyme activities are lower at higher elevations than at lower elevations in the same vertical zone [30]; the lower temperature at high altitudes is the main reason for the increase in environmental stress in this region [31]. Our results for S-SC, S-CAT, and S-ACP enzyme activities were consistent with this pattern, except that the lowest activity of S-UE was observed below 900 m. A study on a high mountain valley zone in Sichuan, China, found that S-UE activity increased and then decreased along the altitude gradient [14], which is consistent with our findings. This indicates that the effect of environmental factors on enzyme activity varies depending on the soil enzyme species [32]. This phenomenon can be explained by the high variability of soil enzyme activity due to the high microbial diversity [23,33].

### 4.2. Vertical Variability in Soil Bacterial Community Structure and Phylogeny in Fanjingshan

The linear regression results showed that altitude gradient had no significant effect on soil bacterial alpha and beta diversity in Fanjingshan. The Chao1 index, Shannon index, and Faith’s PD indicated no differential change in bacterial community richness, bacterial community evenness, and bacterial community phylogeny, respectively, with altitude. However, a tendency remains for soil microbial diversity to increase linearly with increasing elevation. This is consistent with the variation pattern of AM bacteria diversity index observed along the altitude gradient in Fanjingshan [20] and contrary to the summary microbial elevational diversity patterns at the Kohala volcano of Hawaii and Mt. Kilimanjaro, East Africa [34,35]. This indicates that microbial communities in different regions respond differently to environmental changes along an altitude gradient [36].

There were differences in bacterial species richness and diversity changes, with higher species abundance at low- and high-altitude positions. *Acidobacteria* and *Proteobacteria* were identified as the dominant taxa (Figure S1). Previous studies have shown that *Acidobacteria* is a newly discovered group of beneficial bacteria that promote plant growth by regulating ecophysiological effects in soil [37], while *Proteobacteria* can interact with plants to enhance crop resistance to pathogenic fungi [38]. The cooperation between multiple beneficial bacteria promotes local soil health, which is important for maintaining high local biodiversity. Until now, however, soil microbial regulatory mechanisms and the vertical distribution of functional bacterial taxa have not been investigated in Fanjingshan. Further identification of the key functional microflora that play a role in soil microbiology using a metagenomics approach is necessary for future research.

### 4.3. Relationship between Soil Bacterial Community Structure and Soil pH or Enzyme Activity along the Altitude Gradient

Soil pH is often regarded as a determinant of the bacterial community composition and distribution pattern in soil [39]. Despite the small size of our research area, results showed a significant correlation between pH and bacterial diversity or community structure, indicating that some bacterial communities have a relatively narrow ecological niche. This conclusion is supported by the variability exhibited among communities along different elevations [10,40]. In the altitude range below 900 m, bacterial communities were significantly correlated with pH, suggesting that bacterial taxa such as *Delftia*, *Proteus*, and *Xiphinematobacter* distributed on this gradient have narrow ecological niches and are highly sensitive to environmental change.

Hou et al. [41] found that S-ACP and S-UE were positively correlated with microbial quantity, and Shen et al. [42] showed that S-CAT and S-SC activities are susceptible to pH changes. Similarly, in this study, several taxa such as *Pedomicrobium*, *Candidatus Solibacter*, and *Burkholderia* showed significant positive correlations with S-ACP and S-UE (Figure S2), and pH had a positive correlation with S-SC, S-CAT, S-ACP, and S-UE activities. Interestingly, taxa such as *Rhodoplanes*, *Bradyrhizobium*, and *Burkholderia* showed a negative correlation with pH and S-SC, while taxa such as *Pseudomonadaceae* and *Ralstonia* showed no significant correlation with pH and soil enzymes. It has been reported that soil microorganisms were significantly correlated with changes in soil moisture, soil organic matter, effective nutrient content, or vegetation caused by seasonal climate change [43]. This could help to explain our findings. It is necessary to perform long-term dynamic monitoring of microclimate changes in Fanjingshan, which will be considered in our future work.

## 5. Conclusions

In this study, we examined the physicochemical and biological properties (pH and soil enzyme activities), soil bacterial diversity, and community structure across a 2.1-km altitude gradient (comprising samples at six altitude ranges). The S-SC and S-CAT activities showed an overall decreasing trend with altitude, while S-ACP and S-UE activities showed an increasing trend followed by a decreasing trend. Bacterial community diversity showed a linear increasing trend with the highest bacterial abundance in the altitude range of 1500~1800 m. Soil pH was correlated with enzyme activities and bacterial community composition and structure. These results revealed the vertical spatial differences of soil enzyme activities and microbial diversity along the altitude gradient in Fanjingshan. The findings also provide a reference for further research into soil ecosystem service functions.

## Figures and Tables

**Table 1:** Information of sampling points.

No.	Latitude	Longitude	Altitude (m)	Slope (°)	Slope Position	Soil Type
A900-①	27°50′24.1″	108°34′17.9″	703	50	downhill	yellow soil
A900-②	27°49′51.5″	108°34′15.7″	752	40	downhill	yellow soil
A900-③	27°50′42.8″	108°34′26″	714	40	downhill	yellow soil
B1200-①	27°49′55.3″	108°36′11.4″	1107	30	downhill	yellow soil
B1200-②	27°51′42.1″	108°37′3.3″	912	30	downhill	yellow soil
B1200-③	27°49′53.2″	108°36′27.8″	1157	20	mid-slope	yellow soil
C1500-①	27°49′57.4″	108°36′36.2″	1236	35	mid-slope	yellow soil
C1500-②	27°50′2.1″	108°36′44.7″	1348	30	uphill	yellow soil
C1500-③	27°50′2.1″	108°36′55.5″	1478	35	top of the slope	yellow soil
D1800-①	27°50′35″	108°37′4.7″	1504	35	mid-slope	dark yellow-brown soil
D1800-②	27°50′8.1″	108°37′13.0″	1632	40	mid-slope	dark yellow-brown soil
D1800-③	27°50′12.3″	108°37′23.6″	1673	40	base of the slope	dark yellow-brown soil
E2100-①	27°54′25.6″	108°39′14.4″	1848	50	uphill	dark yellow-brown soil
E2100-②	27°54′43.0″	108°39′42.1″	1961	75	uphill	dark mountain dwarf forest soil
E2100-③	27°54′44.2″	108°39′54.9″	1932	60	uphill	dark mountain dwarf forest soil
F2400-①	27°55′5.0″	108°40′57.9″	2169	45	uphill	dark mountain dwarf forest soil
F2400-②	27°55′10.9″	108°41′11.1″	2266	35	top of the slope	mountainous scrub meadow soil
F2400-③	27°55′4.0″	108°41′26.3″	2265	40	top of the slope	mountainous scrub meadow soil

## Data Availability

The datasets used and/or analyzed during the current study are available from the corresponding author on reasonable request.

## Supplementary Materials

The following supporting information can be downloaded at: https://www.mdpi.com/article/10.3390/life12111862/s1, Figure S1: Histogram of bacterial community structure percentage stacking at the phylum level; Figure S2: Heatmap plot of bacterial diversity versus soil pH and soil enzyme activity (*X*-axis is environmental factor, *Y*-axis is species; Figure S3: Results of PCA analysis of MetaCyc pathway; Table S1: Data of soil pH and enzyme activity; Table S2: Spearman’s correlation coefficient analysis of soil pH, enzyme activity and bacterial community richness.
