# Supplementary material for: Spatial Heterogeneity of Soil Bacterial Community Structure and Enzyme Activity along an Altitude Gradient in the Fanjingshan Area, Northeastern Guizhou Province, China
Source: Life (Basel). 2022 Nov 12;12(11):1862. doi: 10.3390/life12111862 (PMC9698955; doi:10.3390/life12111862)
Supplement: Supplementary file 1 [file life-12-01862-s001.zip › figS2.pdf]

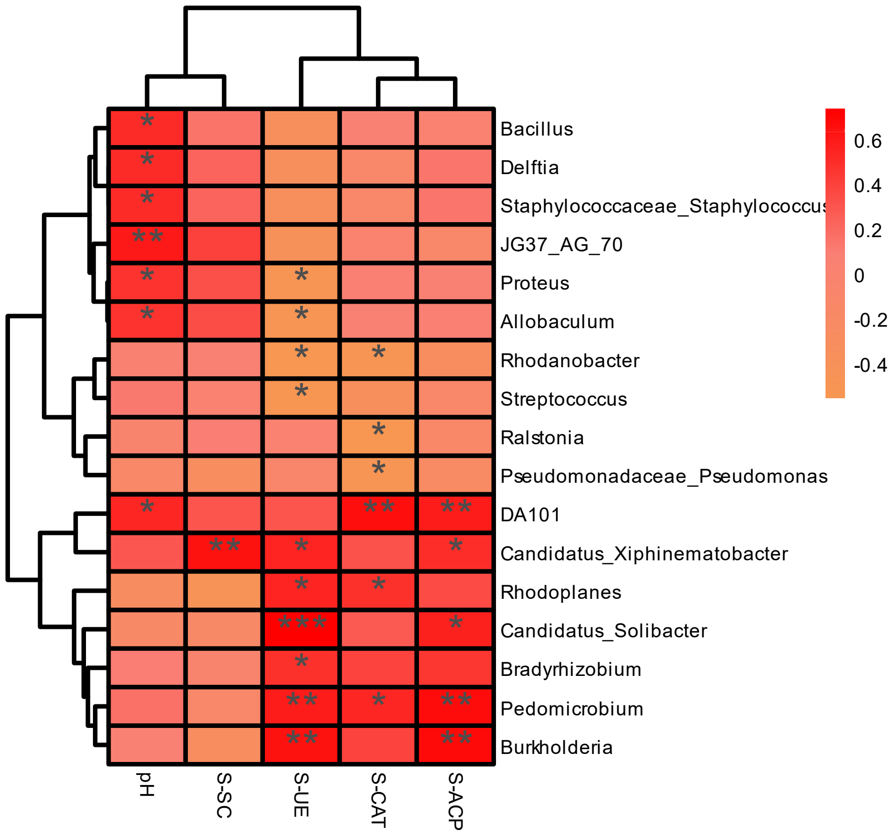

Figure S2. Heatmap plot of bacterial diversity versus soil pH and soil enzyme activity (X-axis is environmental factor, Y-axis is species. R-values and P-values were obtained by rank correlation calculations. R-values are shown in different colors in the plots. P-values are marked with * signs with * represents 0.01≤P<0.05, ** represents 0.001≤P<0.01, * represents P<0.001)**
