# Supplementary material for: Spatial Heterogeneity of Soil Bacterial Community Structure and Enzyme Activity along an Altitude Gradient in the Fanjingshan Area, Northeastern Guizhou Province, China
Source: Life (Basel). 2022 Nov 12;12(11):1862. doi: 10.3390/life12111862 (PMC9698955; doi:10.3390/life12111862)
Supplement: Supplementary file 1 [file life-12-01862-s001.zip › tableS1.pdf]

Table S1 Data of soil pH and enzyme activity

Indicator	Range	Mean±SD	Units
pH	4.56-5.28	4.87±0.05	—
S-SC	22.84-48.35	30.20±2.42	mg/g/d
S-CAT	18.52-25.40	19.56±0.82	umol/g
S-UE	237.78-707.24	464.14±36.68	ug/g/d
S-ACP	44.85-57.18	51.36±1.41	umol/g
