# Supplementary material for: Spatial Heterogeneity of Soil Bacterial Community Structure and Enzyme Activity along an Altitude Gradient in the Fanjingshan Area, Northeastern Guizhou Province, China
Source: Life (Basel). 2022 Nov 12;12(11):1862. doi: 10.3390/life12111862 (PMC9698955; doi:10.3390/life12111862)
Supplement: Supplementary file 1 [file life-12-01862-s001.zip › tableS2.pdf]

Table S2 Spearman's correlation coefficient analysis of soil pH, enzyme activity and bacterial community richness

Indicator	Genus of bacteria	R2 adj	Genus of bacteria	R2 adj
pH	DA101	0.02*	JG37_AG_70	0.01**
	Allobaculum	0.05*	Bacillus	0.03*
	Candidatus_Xiphinematobacter	0.24	Delftia	0.03*
	Rhodoplanes	0.29	Staphylococcaceae_Staphylococcus	0.03*
	Candidatus_Solibacter	0.52	Proteus	0.04*
	Streptococcus	0.61	Pedomicrobium	0.52
	Bradyrhizobium	0.69	Pseudomonadaceae_Pseudomonas	0.6
	Candidatus_Koribacter	0.79	Other	0.77
	Burkholderia	0.8	Rhodanobacter	0.84
	Ralstonia	0.88	Sphingomonas	0.94
S-SC	Candidatus_Xiphinematobacter	0.01**	JG37_AG_70	0.1
	Bradyrhizobium	0.9	Proteus	0.17
	Streptococcus	0.89	Pseudomonadaceae_Pseudomonas	0.25
	Ralstonia	0.7	Delftia	0.35
	Candidatus_Solibacter	0.54	Staphylococcaceae_Staphylococcus	0.35
	Candidatus_Koribacter	0.31	Bacillus	0.55
	Burkholderia	0.26	Pedomicrobium	0.65
	DA101	0.21	Sphingomonas	0.66
	Allobaculum	0.16	Other	0.68
	Rhodoplanes	0.07	Rhodanobacter	0.82
S-CAT	DA101	0.01**	Pseudomonadaceae_Pseudomonas	0.03*
	Ralstonia	0.02*	Pedomicrobium	0.02*
	Rhodoplanes	0.04*	Rhodanobacter	0.03*
	Bradyrhizobium	0.1	Other	0.24
	Burkholderia	0.1	Delftia	0.62
	Streptococcus	0.17	Staphylococcaceae_Staphylococcus	0.62
	Candidatus_Xiphinematobacter	0.19	Sphingomonas	0.76
	Candidatus_Solibacter	0.25	Proteus	0.77
	Candidatus_Koribacter	0.31	Bacillus	0.81
	Allobaculum	0.81	JG37_AG_70	0.98
S-UE	Candidatus_Solibacter	0.01***	Ralstonia	0.97
	Burkholderia	0**	Candidatus_Koribacter	0.36
	Pedomicrobium	0.01**	DA101	0.22
	Bradyrhizobium	0.04*	Sphingomonas	0.93
	Allobaculum	0.03*	Pseudomonadaceae_Pseudomonas	0.69
	Rhodoplanes	0.02*	Bacillus	0.17
	Candidatus_Xiphinematobacter	0.02*	Delftia	0.17

	Streptococcus	0.02*	Staphylococcaceae_Staphylococcus	0.17
	Proteus	0.03*	JG37_AG_70	0.11
	Rhodanobacter	0.02*	Other	0.06
S-ACP	Ralstonia	0.55	Streptococcus	0.67
	Rhodoplanes	0.15	JG37_AG_70	0.66
	Candidatus_Xiphinematobacter	0.04*	Sphingomonas	0.65
	DA101	0.01**	Delftia	0.57
	Candidatus_Solibacter	0.01*	Staphylococcaceae_Staphylococcus	0.57
	Burkholderia	0**	Pseudomonadaceae_Pseudomonas	0.41
	Pedomicrobium	0**	Other	0.38
	Bacillus	0.94	Candidatus_Koribacter	0.25
	Proteus	0.81	Rhodanobacter	0.25
	Allobaculum	0.77	Bradyrhizobium	0.06

Note: * indicates statistical significance at 0.05 level; ** indicates statistical significance at 0.01 level.
