# Supplementary figures and images for: Spatial Heterogeneity of Soil Bacterial Community Structure and Enzyme Activity along an Altitude Gradient in the Fanjingshan Area, Northeastern Guizhou Province, China
Source: Life (Basel). 2022 Nov 12;12(11):1862. doi: 10.3390/life12111862 (PMC9698955; doi:10.3390/life12111862)

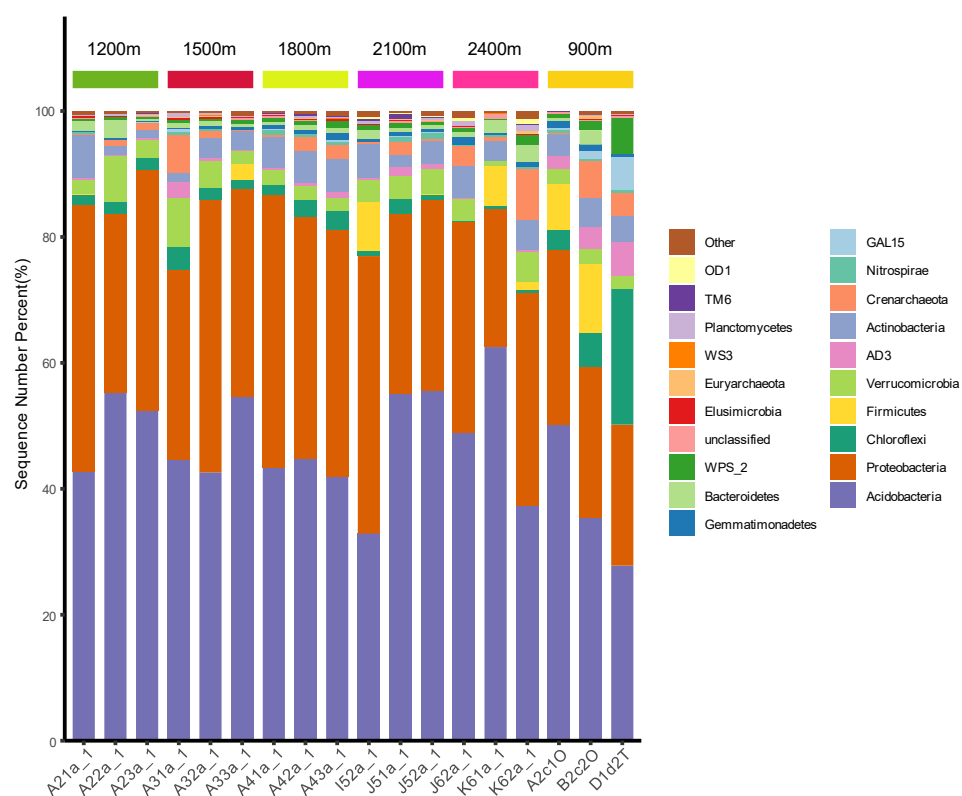

**Figure S1. Histogram of bacterial community structure percentage stacking at the phylum level**

Supplement: Supplementary file 1 [file life-12-01862-s001.zip › figS1.pdf]

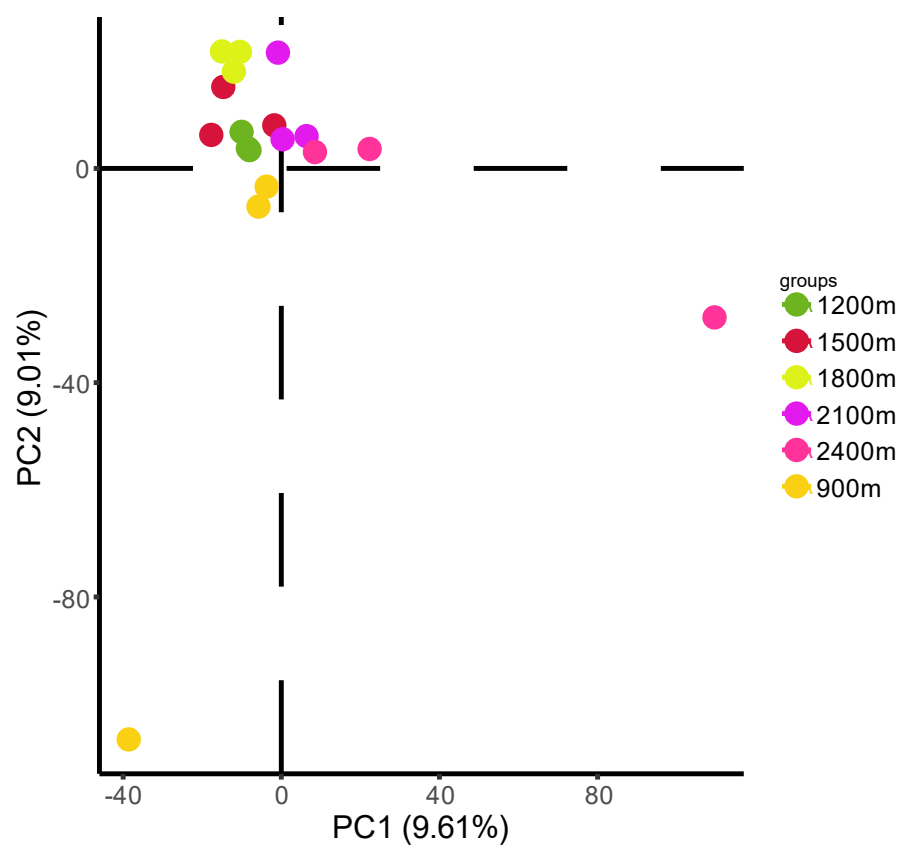

**Figure S3 Results of PCA analysis of MetaCyc pathway**

Supplement: Supplementary file 1 [file life-12-01862-s001.zip › figS3.pdf]
